# On Neurorights

**DOI:** 10.3389/fnhum.2021.701258

**Published:** 2021-09-24

**Authors:** Marcello Ienca

**Affiliations:** ^1^College of Humanities, École Polytechnique Fédérale de Lausanne (EPFL), Lausanne, Switzerland; ^2^Department of Health Sciences and Technology, ETH Zürich, Zurich, Switzerland

**Keywords:** neurorights, neuroethics, neurolaw, human rights, science policy

## Abstract

In recent years, philosophical-legal studies on neuroscience (mainly in the fields of neuroethics and neurolaw) have given increasing prominence to a normative analysis of the ethical-legal challenges in the mind and brain sciences in terms of rights, freedoms, entitlements and associated obligations. This way of analyzing the ethical and legal implications of neuroscience has come to be known as “neurorights.” Neurorights can be defined as the ethical, legal, social, or natural principles of freedom or entitlement related to a person’s cerebral and mental domain; that is, the fundamental normative rules for the protection and preservation of the human brain and mind. Although reflections on neurorights have received ample coverage in the mainstream media and have rapidly become a mainstream topic in the public neuroethics discourse, the frequency of such reflections in the academic literature is still relatively scarce. While the prominence of the neurorights debate in public opinion is crucial to ensure public engagement and democratic participation in deliberative processes on this issue, its relatively sporadic presence in the academic literature poses a risk of semantic-normative ambiguity and conceptual confusion. This risk is exacerbated by the presence of multiple and not always reconcilable terminologies. Several meta-ethical, normative ethical, and legal-philosophical questions need to be solved in order to ensure that neurorights can be used as effective instruments of global neurotechnology governance and be adequately imported into international human rights law. To overcome the shortcomings above, this paper attempts to provide a comprehensive normative-ethical, historical and conceptual analysis of neurorights. In particular, it attempts to (i) reconstruct a history of neurorights and locate these rights in the broader history of idea, (ii) outline a systematic conceptual taxonomy of neurorights, (iii) summarize ongoing policy initiatives related to neurorights, (iv) proactively address some unresolved ethico-legal challenges, and (v) identify priority areas for further academic reflection and policy work in this domain.

## Introduction: A Brief History of Neurorights

Over the last two decades, technological progress in the field of neuroscience and neuroengineering, in conjunction with the translation of neurotechnological innovation into extra-clinical sectors (e.g., the judiciary, the military, and the consumer industry), has resulted in growing public interest and academic reflection on the ethical and societal implications of technologies that intercommunicate with the human brain. *Neurotechnology* is the umbrella term typically used to describe this broad and heterogenous spectrum of methods, systems and instruments that establish a direct connection pathway to the human brain through which neuronal activity can be recorded and/or influenced. As a result of this growing academic and public interest, whole new disciplines and subdisciplines have emerged. These include neuroethics and neurolaw. *Neuroethic*s was defined by Safire as “the examination of what is right and wrong, good and bad about the treatment of, perfection of, or unwelcome invasion of and worrisome manipulation of the human brain” (Safire, 2002). The term *neurolaw* was first coined by Sherrod Taylor in the early 1990s to denote the growing area of collaboration between neuropsychologists and lawyers in the criminal justice system (Taylor et al., 1991). In the subsequent decades, the purview of neurolaw was expanded to envelop the whole area of intersection between neuroscience and the law (Shen, 2016). The foundation of the International Neuroethics Sociey (INS), which was the byproduct of a meeting held in Asilomar (United States) in 2006, marked a milestone toward the institutionalization of neuroethics and neurolaw as academic disciplines. Three lustra later, the INS constitutes the largest academic society committed to studying the social, legal, ethical and policy implications of advances in neuroscience.

Throughout the 1990s and early 2000s, the dominant discourse in public opinion and academic reflection on neuroethics and neurolaw focused mainly on four main thematic families:

A. The ethical permissibility of cognitive enhancement *via* nootropics (Farah et al., 2004; Turner and Sahakian, 2006);
B. The philosophical-legal implications of the neuroscience of free will with special focus on the notions of moral responsibility and legal culpability (Pereboom and Caruso, 2002; Moreno, 2003; Fins, 2004);
C. The ethics of neuroimaging, especially with regard to mind reading (Farah, 2002; Illes et al., 2003, 2004); and
D. The validity and permissibility of neuroscientific evidence in court (Reider, 1998; Moreno, 2003; Zeki et al., 2004).

Since the beginning of the current century, a fifth and complementary area of neuroethical and neurolegal enquiry has emerged, which has begun to look at ethical-legal challenges in neuroscience and neurotechnology in terms of high-level normative principles, such as rights, entitlements, and associated duties. This way of analyzing the ethical and legal implications of neuroscience has come to be known as “neurorights.” Neurorights can be defined as the ethical, legal, social, or natural principles of freedom or entitlement related to a person’s cerebral and mental domain; that is, the fundamental normative rules for the protection and preservation of the human brain and mind.

This paper aims to take stock of the emerging debate on neurorights. In particular, it attempts to trace a history of neurorights and locate these rights in the broader history of ideas, outline a systematic conceptual taxonomy of neurorights, summarize ongoing policy initiatives related to neurorights, proactively address some unresolved ethical-legal challenges, and, finally, identify priority areas for further academic reflection and policy work in this field.

### From Neuroethics to Neurorights

A pioneering step toward neurorights was marked by Boire’s (2001) and Sententia’s (2004) work on the notion of “cognitive liberty” in the early 2000s. Sententia (2004, p. 227) defined cognitive liberty as “the right and freedom to control one’s own consciousness and electrochemical thought process.” It should be noted that this field of ethical-legal enquiry has emerged in full continuity with the dominant debates in neuroethics and neurolaw listed above. Boire (2001), for instance, developed his reflections on cognitive liberty in a dialog with ongoing debates on the ethics of neuroimaging and mind reading. In a similar fashion, Sententia (2004) developed her definition and normative analysis of cognitive liberty by taking stock of the ongoing neuroethical debate on cognitive enhancement.

The point of departure of their analyses, however, is normative-theoretical in nature: both authors posited that the concept of cognitive liberty should be interpreted not simply as a neurophilosophical description or a moral desideratum but as a “fundamental right” (Sententia, 2004, p. 223). In particular, Sententia (2004, p. 227) argued that advances in neurotechnology require a high-level analysis that is contextual to “those individual rights embedded in our democratic constitution” and posited that cognitive liberty “is the necessary substrate for just about every other freedom.” In the 2010s, this right-based view of cognitive liberty was further expanded by Farahany’s (2012) doctrinal analysis of, respectively, the Fourth Amendment to the United States Constitution and the Self Incrimination Clause of the Fifth Amendment. Further, it was reaffirmed by Bublitz’s (2013) thesis that the use of “mind-interventions outside of the therapeutic contexts” should urge the law to recognize cognitive liberty (which he also called “mental self-determination”) as a “basic human right” which “guarantees an individual’s sovereignty over her mind.” While none of the above-mentioned authors used the term, this body of scholarship laid the groundwork for the emerging area of enquiry at the intersection of neuroethics and neurolaw that is now increasingly known to the public as “*neurorights*.” This branch of enquiry has introduced a new angle from which we can look at the ethical-legal challenges in the mind and brain sciences, namely in terms of rights (be they legal rights or rights in the philosophical sense), freedoms, entitlements and associated obligations.

The term “*neuroright*” was first introduced by Ienca and Andorno (2017a, b) in April 2017 in an ancillary article to their ethical-legal analysis of human rights in the age of neuroscience and neurotechnology. Those authors conducted a parallel analysis of, respectively, emerging trends in neurotechnology and human rights provisions related to the protection of the human brain and mind contained in existing human rights instruments, such as the United Nation’s Universal Declaration of Human Rights (UDHR) (1948), the European Union’s Charter of Fundamental Rights (2000) and the UNESCO’s Universal Declaration on Bioethics and Human Rights (2005). Their comparative doctrinal analysis concluded that existing human rights are necessary but may not be normatively sufficient to respond to the emerging issues raised by neurotechnology. For this reason, the authors made the case that “the possibilities opened up by neurotechnological developments and their application to various aspects of human life will force a reconceptualization of certain human rights, or even the creation of new rights to protect people from potential harm” (Ienca and Andorno, 2017b). In particular, they identified four new neuro-specific rights, hence called *neurorights*, that, in their view, may offer suitable conceptual ground for normative analysis in this domain: the right to cognitive liberty (which they interpreted in agreeance with Sententia and Bublitz), the right to mental privacy, the right to mental integrity, and the right to psychological continuity. This article sparked a debate on public media and in the academic community. Among others, Cascio (2017) endorsed the proposal but questioned whether neurorights should be seen as legal rights of the mind or of the person. Further, he critically discussed the limits of neurorights (e.g., in the case of minors). More optimistically, Pizzetti (2017) argued, in a letter to the UNESCO Chair of Bioethics, that the four neurorights identified by Ienca and Andorno may constitute the building blocks of a “Universal Declaration on Neuroscience and Human Rights.” In contrast, Nawrot (2019) criticized the proposal and casted doubts on the potential of neurorights to “reconcile the technological infiltration into our interior castle” (figurative for the human brain and mind) with the concept of “freedom of thought” and the “foundation of a democratic state ruled by law.”

Around the same time Sommaggio and Mazzocca (2020) further investigated the relationship between human rights and cognitive liberty. They concluded that the notion of cognitive liberty provides the necessary conceptual ground for building “a human neuro-rights declaration.”

About half a year later, a paper published in the journal *Nature* and authored by a team of 25 researchers coordinated by Rafael Yuste and Sara Goering reignited and amplified the debate on neurorights (Yuste et al., 2017). The authors identified four areas of concern associated with neurotechnology and AI, namely privacy and consent, agency and identity, augmentation, and bias. For each of those areas of concern, they argued, “clauses protecting such rights (called neurorights)” should be added to international treaties (ivi). This article was extremely influential in the public opinion. By shifting the focus of the neurorights discourse from ethical-legal analysis to policy advocacy, this proposal exerted a great impact on nation-level legislative reforms, most notably in the Republic of Chile. Although the semantics, theoretical justification and normative demarcation of these rights were not addressed in the original article, this proposal was further elaborated in more detail a few years later by Yuste et al. (2021) as well as Goering et al. (2021). In addition, Yuste’s advocacy work led first to the creation of the Neurorights Initiative at Columbia University—the first institutional think-thank on neurorights—and then, in collaboration with European and North American partners, to the Neurorights Network, i.e., the first international network of scholars working on neurorights, whose membership currently spans four continents.

## Historical Antecedents of Neurorights

Neurorights did not emerge out of thin air. In the history of philosophy and political-legal thought, several conceptual constructs can be identified as historical antecedents and conceptual foundations of neurorights. In particular, we can identify three main conceptual families: freedom of thought and conscience, the right to privacy and the right to mental integrity.

### Freedom of Thought and Conscience

The thesis that the human mind and the cognitive processes it enables are free is virtually ubiquitous in the history of ideas. One of the earliest records of this idea dates to the Maurya Dynasty that ruled almost all of the Indian subcontinent in the third century BC. In particular, in the second half of the century, the Indian emperor Ashoka the Great issued edicts promoting respect for “freedom of conscience” (Luzzatti, 2006). A couple of centuries later, Paul of Tarsus discussed, in his first letter to the Corinthians, the extent to which someone’s freedom [in Ancient Greek *“eleutheria*”] should be judged by another’s conscience [*suneideseos*] 10:29 (Collins and Harrington, 1999). In Christian philosophy, the notion of freedom of conscience was often entwined with the notion of *liberum arbitrium*, which is usually translated into English as “free will.” However, while freedom of conscience constituted a normative principle (typically related to a political commitment to religious tolerance), free will was originally conceptualized as a descriptive ontological statement about the lack of necessity of human will. This descriptivist account of free will was rooted in the late ancient Greek philosophy, especially among the Stoics. The Stoic philosopher Epictetus, for instance, regarded it as a “fact that nothing hindered us from doing or choosing something that made us have control over them” (Long, 2002).

During the Renaissance, several concepts related to freedom of conscience emerged. For instance, in the seventeenth century, the Puritan minister and theologian Roger Williams coined the notion of “soul liberty,” that is, the idea that God had endowed human beings with the inborn right to make choices in matters of faith (Gaustad, 2001). This notion later evolved into the notion of “freedom of religion” or “religious liberty,” which is currently protected by the UDHR. Around the same time, poet John Milton used the expression “freedom of the mind” to indicate the right and ability of people to protect their minds from external interference (Milton, 1791). Milton was among the first thinkers to introduce the idea that the human mind is the last refuge of personal freedom and self-determination. In the nineteenth century, this idea was further expanded by Mill (1859, p. 12), who argued that “[o]ver himself, over his own body and mind, the individual is sovereign.” Moving from moral philosophy to modern literature, this notion of freedom of the mind was taken up, in the twentieth century, by Woolf (1929), who famously wrote: “There is no gate, no lock, no bolt that you can set upon the freedom of my mind.” This view of the mind as the ultimate locus of personal freedom has been highly influential for the debate on neurorights. For example, Sententia (2004) implicitly referred to this tradition by arguing that “the right and freedom to control one’s own consciousness and electrochemical thought processes is the necessary substrate for just about every other freedom.”

Freedom of thought in the normative sense is protected by the Universal Declaration of Human Rights (UDHR), which is legally binding on member states of the International Covenant on Civil and Political Rights (ICCPR). Particularly, the right to freedom of thought is listed under Article 18, which states the following:

*Everyone has the right to freedom of thought, conscience and religion; this right includes freedom to change his religion or belief, and freedom, either alone or in community with others and in public or private, to manifest his religion or belief in teaching, practice, worship and observance.*

The UDHR establishes a *prima facie* link between freedom of thought and freedom of religion. In addition, the United Nations Human Rights Committee (UNHRC) emphasized that the scope of the right to freedom of thought is “far-reaching and profound; it encompasses freedom of thoughts on all matters” (United Nations Human Rights Committee (UNHRC), 1993). The UNHRC has also clarified that the “the freedom of thought, conscience, religion or belief” should be distinguished from “the freedom to manifest religion or belief” (United Nations Human Rights Committee (UNHRC), 1993) stated that the UDHR “does not permit any limitations whatsoever on the freedom of thought and conscience or on the freedom to have or adopt a religion or belief of one’s choice. These freedoms are protected unconditionally” (ivi). This interpretation would make freedom of thought and conscience one of the very rare *absolute rights*, as opposed to *relative rights*, as these two rights are valid unconditionally and independently of contextual variables.

In the neurorights debate, Ienca and Andorno (2017b) have further emphasized the distinction between freedom of thought and the freedom to manifest thought or belief. They argued that cognitive liberty protects the sphere of thought even prior to any externalization or manifestation of thought through speech, writing, or behavior. As such, they argued, cognitive liberty is chronologically antecedent to any other freedom (Ienca and Andorno, 2017b) and complementary to notions, such as freedom of speech, freedom of the press and freedom of assembly.

In the United States, the protection of freedom of thought is frequently associated with the First Amendment (Richards, 2015). Although the Amendment does not mention freedom of thought explicitly, U.S. courts have explicitly referred to a “First Amendment right to freedom of thought” (Doe v. City of Lafayette, Indiana, 2003). Furthermore, the U.S. Supreme Court has stated that “at the heart of the First Amendment is the notion that an individual should be free to believe as he will” (Abood v. Detroit Board of Education, 1977).

Many authors have considered freedom of thought as a precursor and progenitor of other freedoms, such as freedom of religion and freedom of expression. This fundamental role of freedom of thought as a substratum for other freedoms was recognized by, among others, US Supreme Court Justice Benjamin Cardozo whose reasoning in Palko v Connecticut (1937) was as follows: “Freedom of thought…is the matrix, the indispensable condition, of nearly every other form of freedom. With rare aberrations a pervasive recognition of this truth can be traced in our history, political and legal” (Polenberg, 1996). Sententia’s argument that cognitive liberty should be considered the substratum of all other freedoms can be subsumed into this legal-philosophical tradition. By virtue of this precursor nature, freedom of thought can be considered axiomatic for the other freedoms, since these freedoms are in no way required for it to operate and exist.

### Privacy

Although the right to privacy has been present *in nuce* in the notions of freedom and personal autonomy, the first consistent conceptualization of the modern right to privacy dates back to a seminal article, published in 1890, by Warren and Brandeis. In this article, privacy was conceptualized as “a right to be let alone” (Brandeis and Warren, 1890). At the time the article was written, Warren and Brandeis’ main concern was the growing interest of the print media in gossiping and revealing personal information about individuals without their consent, which they regarded as an invasion of a person’s private sphere. This specific instance of privacy was further developed by Westin and other authors into the broader notion of “information privacy,” i.e., the control over information about oneself. According to Westin (1968), information privacy can be defined as everyone’s claim to determine for themselves when, how, and to what extent personal information is communicated to others.

International human rights law formally recognizes a right to privacy. The Universal Declaration of Human Rights (UDHR) states that “no one shall be subjected to arbitrary interference with his privacy, family, home or correspondence, nor to attacks upon his honor and reputation. Everyone has the right to the protection of the law against such interference or attacks” (Article 12). Similarly, the 1950 European Convention on Human Rights (ECHR) stipulates that “everyone has the right to respect for his private and family life, his home and correspondence” (Article 8 para 1) and specifies that this right involves “protection against telephone tapping, collection of private information by a state’s security services and publications infringing privacy” (Article 8).

In today’s digital world, the right to privacy has become relevant to whole new domains and methods of information processing that were unthinkable at the time of Warren and Brandeis or even of the UDHR; among them: the brain-mind sphere and data processing techniques aimed at revealing information about a person’s mental processes or neurological health. This category of privacy challenges includes both the predictive analysis of primary neural data, such as brain recordings and inferences based on secondary data (e.g., phenotypic, or behavioral data) through techniques, such as affective computing. For example, Yuste et al. (2017) argued that “an extraordinary level of personal information can already be obtained from people’s data traits” and argued that “citizens should have the ability—and right—to keep their neural data private.” Based on similar considerations—with special regard to the security vulnerabilities of neurodevices, the nature of neural data and the inferential potential of advanced data analytic techniques—Ienca and Andorno (2017b) proposed to evolutionarily reinterpret the right to privacy and proposed the recognition of a “right to mental privacy” which would explicitly protect individuals against the unconsented intrusion by third parties into their mental information (be it inferred from their neural data or from proxy data indicative of neurological, cognitive, and/or affective information) as well as against the unauthorized collection of those data. A conceptually similar right to mental privacy was also proposed by Yuste et al. (2017). All these authors established an intimate relationship between the notion of privacy as applied to the mental domain and freedom of thought. Historically observed, this relationship between mental privacy and freedom of thought had been already investigated, in the early twentieth century, by historian J.B. Bury. In his famous “A History of Freedom of Thought,” he argued that “a man can never be hindered from thinking whatever he chooses as long as he conceals what he thinks” (Bury, 1913, p. 1). This suggests that exercising one’s right to mental privacy—and thereby concealing one’s own thoughts—is necessary to fully exercise one’s own right to freedom of thought.

A somewhat surprising historical antecedent of the right to mental privacy is reported by philosopher and statesman Francis Bacon, who chronicled that Queen Elisabeth I revoked a thought censorship law in the late sixteenth century, because, allegedly, she did “not [like] to make windows into men’s souls and secret thoughts” (Brimacombe, 2000).

### Mental Integrity

While freedom of thought protects the human mind from external interference and the right to privacy protects personal information (including mental information) from external intrusion, other normative principles protect the human mind from harm. In the history of ideas, the ethical principle of “non-maleficence” is the most comprehensive conceptual construct postulating the protection of a person’s integrity and the avoidance of harm.

The moral obligation ‘‘to abstain from doing harm’’ is already present in some early versions of the Hippocratic Oath and is widely reported throughout the medical deontology literature. This moral obligation was later reformulated in the Latin maxim ‘‘*primum non-nocere*,’’ that is ‘‘first do no harm’’.^1^ Although the principle of avoiding harm embedded in the ethos of medicine and biomedical research, the characterization of harm is not always semantically straightforward. The medical ethics literature classifies harm according to its magnitude, severity, duration, and reversibility (Meslin, 1990). Further, it distinguishes various types of harm depending on the personal sphere or capability affected by the harmful intervention. These include physical, psychological, and socio-economic harm. However, the separation of physical and psychological harm is questionable as it implicitly assumes a dualistic ontology of the person (body vs. mind). Further, it has been observed that novel forms of harm enabled by emerging technologies may not easily fit into this classification (Hayes, 2017; Favaretto et al., 2020).

Preventing psychological harm, such as harm from psychological abuse, is one eminent historical antecedent of neurorights, especially of the right to mental integrity. The occurrences of the right to mental integrity in the history of ideas are relatively scant. In the early 1970s, Welford used the notion of mental integrity as a criterion for demarcating the ethical boundary between the obligation to provide life-maintaining treatment and unreasonable therapeutic obstinacy, especially among the terminally ill, the senile and severely deficient children (Welford, 1970). The right to mental integrity—together with its corollary, namely physical integrity— is protected under the EU’s Charter of fundamental rights, whose Article 3 states that “everyone has the right to respect for his or her physical and mental integrity.” The Charter focuses in particular on four requirements: free and informed consent, the non-commercialization of body elements, and the prohibition of eugenic practices and human reproductive cloning. No explicit reference, however, is made to neurotechnology-related practices or specific harms caused by malevolently interfering with a person’s neuropsychological sphere.

Mental integrity also has affinities with normative principles for the protection of people who have a mental disorder. Notably, Article 7 of the Council of Europe’s Oviedo Convention (“Protection of persons who have a mental disorder”) defines the conditions under which people who have a mental disorder may or may not be subjected to an intervention without their consent. Finally, mental integrity offers potentially suitable normative ground for protecting people from discrimination based on their neural and/or mental characteristics, a type of discrimination called “neurodiscrimination” (Ienca and Ignatiadis, 2020).

### Personal Identity

In philosophy, particularly in the philosophy of mind, personal identity is the unique identity of a person−who is considered subject of consciousness−over time. Personal identity is often referred to as the set of properties that define someone as an individual person or make someone the person he or she is, and which distinguish them from others. Consequently, the notion of personal identity often presupposes a notion of personhood, i.e., the status of being a person as opposed to a non-person. Most philosophers interpret personhood in terms of a certain set of mental properties (Baker, 2000). However, there is ample disagreement with regard to determining which mental properties are constitutive of personhood. Candidates include self-awareness, proprioception, and the capacity to suffer. Another frequently invoked requirement of personhood is persistence, namely the fact that personhood persists from one time to another. The issue of persistence of personal identity is addressed by so-called psychological continuity theories of personal identity. This bundle of theories defines personal identity in terms of overlapping chains of psychological connections that are appropriately caused. These psychological connections may involve memories or other cognitive or affective states such as, for instance, an intention and the action carried out by such intention, or the relationship between different temporal portions of a continuing belief.

In legal theory, the right to personal identity is everyone’s right to form an individual identity, develop a conscience, and protect such individual identity and conscience from external limitations, manipulation, or erasure. It is believed that the right to personal identity begins with the right to life, since it is only through existence that individuals can cultivate their identity. This right is recognized in international law through a range of declarations and conventions. For example, the European Court of Human Rights (ECHR) interpreted Article 8 of the European Convention on Human Rights as to include ‘‘personal identity’’ within the meaning of ‘‘private life,’’ which is explicitly protected from unwanted intrusion by third parties.^2^

## Demarcating the Conceptual Ontology of Neurorights

It is notable that reflections on neurorights have received extensive coverage in the mainstream media. However, the presence of such reflections in the academic literature is still relatively scarce. Although it has rapidly become a mainstream topic within neuroethical discourse, this area of study is still in a germinal stage of theoretical maturity. This is attested by the fact that the number of publications about neurorights in mainstream media largely outnumbers the quantity of scholarly publications on this topic.^3^

While the prominence of the debate on neurorights in public opinion is crucial to ensure public engagement and democratic participation in deliberative processes on this issue, its relative sporadic nature in the academic literature raises a risk of semantic-normative ambiguity and conceptual confusion. This risk is exacerbated by the presence of multiple and not always reconcilable terminologies. Above all, several meta-ethical, normative ethical and legal questions need to be solved. For these reasons, in this section we will try to provide a systematic classification of the neuro-rights proposed so far. Finally, in the next section, we will discuss the main conceptual issues still open.

**FIGURE 1:**
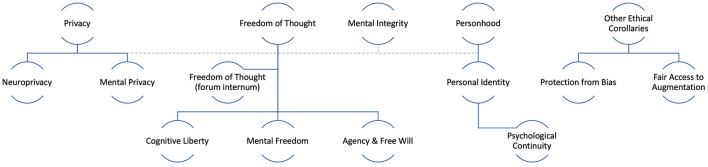
A taxonomy of neurorights.

First of all, let us consider the very notion of “neurorights.” Neurorights can be defined as the ethical, legal, social or natural principles of freedom or entitlement relating to a person’s cerebral and mental domain; that is, the fundamental normative rules for the protection and preservation of the human brain and mind. Consequently, neuroright studies are a subfield of neuroethical and neurolegal inquiry that deals with the ethical, legal, social or natural principles of freedom or entitlement related to a person’s cerebral and mental domain; that is, the fundamental normative rules for the protection and preservation of the human brain and mind. We can identify at least five families of neurorights, depending on the normative ethical principles from which they derive: derivatives of freedom of thought, derivatives of privacy, derivatives of mental integrity, derivatives of personal identity and other ethical corollaries. Figure 1 provides a visual taxonomy of neurorights.

### Derivatives of Freedom of Thought

Four neurorights conceptually derived from freedom of thought have been proposed in the literature. These are: cognitive liberty, the right to agency and free will, mental freedom, and freedom of thought itself.

As we have seen earlier, cognitive liberty was a precursor to the neuro-rights debate. Albeit there are differences in its formulation, there is a general consensus in the literature that cognitive liberty entails a person’s autonomous, unhindered control over their mind. This is well-captured by Bublitz’s (2013) use of cognitive liberty as a synonym for “mental self-determination.” According to Bublitz (2013), this right comprises two fundamental and intimately related principles: (a) the right of individuals to freely use emerging neurotechnologies; (b) the protection of individuals from the coercive and unconsented use of such technologies. In other words, cognitive liberty is the principle that guarantees “the right to alter one’s mental states with the help of neurotools as well as to refuse to do so” (Bublitz, 2013, p. 234). Analogously, Ienca and Andorno pointed out that cognitive liberty is a “complex right which involves the prerequisites of both negative and positive liberties” in the sense of Berlin (1969): the negative liberty of making choices about one’s own cognitive domain in absence of external obstacles, barriers or prohibitions; the negative liberty of exercising one’s own right to mental integrity in absence of external constrains or violations; and finally, the positive liberty of having the possibility of acting in such a way as to take control of one’s mental life (Ienca and Andorno, 2017b). While there is general agreement on the basic premises of cognitive liberty, there is disagreement with regard to its domain of application. Most definitions, including Bublitz’s definition above, limit the purview of cognitive liberty only to alterations of mental states induced by “neurotools” or “neurotechnologies.” In the same article, Bublitz proposes an even narrower definition of cognitive liberty which is restricted to the use of neurotechnology for the purpose of neuroenhancement (p. 233). This definition, accordingly, seems to exclude alterations of mental states that do not enhance brain function (e.g., those that diminish it or cause qualitative instead of qualitative changes). In contrast, Ienca and Vayena (2018) have proposed a broader and medium-independent definition which also envelops unintended alterations of mental states induced by non-neurotechnologies, such as *via* social media and online manipulation, irrespective of whether they result in enhancement, diminishment or no change in brain function.

A “right to agency, or the freedom of thought and free will to choose one’s own actions” has been advocated by Yuste et al. (2021). Although these authors use these three notions as synonyms, as indicated by the disjunctive logical operator “or,” agency, freedom of thought and free will typically denote quite distinct concepts. Agency, as it is widely discussed in the philosophy of action literature, denotes the exercise or manifestation of an agent’s capacity to act. Free will, as we have seen, is an ontological thesis related to the capacity of agents to choose between different courses of action without hindrance. In other words, agency pertains to the domain of action. Free will, in contrast, pertains to the domain of cognition, decision-making in particular. Most importantly, both agency and free will are typically conceptualized as abilities or dispositions. They are descriptive in nature, not normative. Deriving normativity from these descriptive statements requires inferring entitlements and obligations from abilities and dispositions. The logic of such inference, however, remains currently unclear. Finally, as observed by Munoz (2019), “free will is a multidimensional concept that poses several unsolved philosophical problems.”

Mental freedom is used seldom in the literature. Repetti (2018) used mental freedom to outline his Buddhist theory of free will. A powerful use of mental freedom (which he also calls “freedom of mind”) in the context of neurorights is provided by Bublitz (2016) who described it as the “conscious control over one’s mind.” He argued that mental freedom should be ranked among the most important legal and political freedoms (ivi). It is not clear, however, whether “mental freedom,” in Bublitz’s sense, should be interpreted as a synonym for cognitive freedom or as a distinct concept.

Finally, some authors have argued that the very notion of freedom of thought offers suitable normative ground to address the human rights challenges raised by novel neurotechnologies (Lavazza, 2018). Adopting freedom of thought as the normative foundation of a person’s autonomous control over her mind is advantageous from a conceptual parsimony perspective. The Occam’s razor principle or law of parsimony postulates that “entities should not be multiplied without necessity” (Schaffer, 2015). Since freedom of thought is already enshrined in international human rights law and widely discussed in legal philosophy, it would be *ceteris paribus* more parsimonious to adopt this normative terminology compared to multiplying the number of normative entities by introducing cognitive liberty, mental freedom and the rights to agency and free will. In that event, however, it should be clarified that “the protection of a person’s self-determination over her mind should comprise the entire forum internum (Bublitz, 2015), that is all mental states or capacities and there-with cognitive, emotional and conative phenomena, either conscious or unconscious.” As Ienca and Andorno have pointed out, freedom of thought is the fundamental justification for related freedoms, such as freedom of choice, freedom of speech, freedom of press, and freedom of religion. An evolutionary interpretation of this right should focus on the protection not only of externalizations of thought but thought itself.

### Derivatives of Privacy

Unlike the derivatives of freedom of thought, the neurorights originating from the right to privacy seem to be characterized by a much greater degree of conceptual and terminological agreement. Mental privacy is the expression generally used to denote people’s right against the unconsented intrusion by third parties into their brain data as well as against the unauthorized collection of those data (Shen, 2013; Ienca and Andorno, 2017a,b; Yuste et al., 2021). Yuste et al. (2021) argued that mental privacy is not only a right but also an ability, i.e., “the ability to keep thoughts protected against disclosure.” The relationship between mental privacy and the general right to private life is debatable. Ienca and Andorno argued that the special nature of brain information, which relates directly to one’s inner mental life and personhood, and the distinct way in which such data are obtained, requires attaching additional specifications to the current privacy frameworks. They argued that mental privacy should protect brainwaves not only as data but also as data generators or sources of information. In addition, a right to mental privacy would protect not only conscious brain data but also data that are not (or are only partly) under voluntary and conscious control. Further, it should guarantee the systemic protection of brain information. This would contribute to protecting people’s right against illegitimate access to their brain information and to preventing the indiscriminate leakage of brain data across the infosphere.

Another concept frequently used to address people’s moral entitlement to protect their brain information is neuroprivacy. While “mental privacy” aims at protecting mental information, however collected or inferred, neuroprivacy relates specifically to the protection of neural data—also called neurodata or brain data (Hallinan et al., 2014; Ienca, 2015; Wolpe, 2017).

### Derivatives of Freedom of Mental Integrity

A strong conceptual convergence is also recognizable with regard to mental integrity. As we saw earlier, the right to mental integrity is enshrined in the EU’s Charter of Fundamental Rights (Article 3). However, there are differences on how this right is interpreted. Ienca and Andorno (2017b) defined the neuroright to mental integrity as the right of individuals to be protected from illicit and harmful manipulations of their mental activity. In contrast, Lavazza (2018) defines it as “the individual’s mastery of his mental states and his brain data so that, without his consent, no one can read, spread, or alter such states and data in order to condition the individual in any way.” The conceptual difference here is substantial. While Lavazza considers mental integrity as synonymous with cognitive liberty and/or freedom of thought, Ienca’s and Andorno’s definition establishes a necessary logical relationship between mental integrity and the protection from harm related to someone’s neural and/or mental domain. In the first case, it would follow that mental integrity is a substitute for cognitive liberty and freedom of thought. In the latter, it is complementary to them.

### Derivatives of Personal Identity

Some authors have argued for the recognition of a fourth family of neurorights related to the protection of personal identity. Borrowing the terminology from the psychological-continuity account of personal identity (Van Inwagen, 1997), Ienca and Andorno (2017a) called this right “psychological continuity” and described it as the right to preserve “people’s personal identity and the continuity of their mental life from unconsented external alteration by third parties.” Yuste et al. (2021) in contrast, advocated a “right to identity,” which they described as “the ability to control both one’s physical and mental integrity.” While psychological continuity, in its original formulation, has thematic affinities to cognitive liberty and freedom of thought (of which it may be a subtype), the right to identity in Yuste’s sense appears to be a prerequisite for physical and mental integrity.

### Other Ethical Corollaries

Finally, some authors have proposed the recognition of rights that are not directly related to the protection of the mental domain but rather to the promotion of some socio-technical requirements that are instrumentally necessary for the realization of the rights above. Two of these normative ethical corollaries have been proposed: the right to fair access to mental augmentation and the right to protection from algorithmic bias. The former is defined by Yuste et al. (2021) as “the ability to ensure that the benefits of improvements to sensory and mental capacity through neurotechnology are distributed justly in the population” (p. 160–161); the latter is defined by the same authors as “the ability to ensure that technologies do not insert prejudices” (ivi). As such, the right to fair access to mental augmentation appears to be a prerequisite for cognitive liberty in the positive sense. In contrast, the right to protection from algorithmic bias appears to be a prerequisite for the right to mental integrity as it protects from the spectrum of harms generated by algorithmic bias, first and foremost algorithmic discrimination. It is worth noting that unlike all other neuroright candidates described above, the right to protection from algorithmic bias can be and has been advocated in domains that unrelated to the mental and/or neurocognitive sphere, such as fintech, web applications, chatbots, and automation (Garcia, 2016).

## Ongoing Policy Developments

Several governmental, intergovernmental and non-governmental actors are currently actively involved in the governance of neurotechnology. Some of these governance initiatives have included the promotion of neurorights, or the consideration thereof, within their agenda. A first important step was marked in 2019, when the Council of the Organization of Economic Development and Cooperation (OECD) adopted a “Recommendation on Responsible Innovation in Neurotechnology,” which set the first international standard in neurtechnology governance (OECD-Council, 2019). While the OECD Recommendation is primarily focused on responsible governance by neurotechnology industry actors, it features provisions on neurorights, such as mental privacy and cognitive liberty. Other international organizations are putting neurorights at core of their governance strategies. For example, the Council of Europe has launched a 5-year Strategic Action Plan focused on Human Rights and Technologies in Biomedicine, which contains a module on the assessment of the relevance and sufficiency of the existing human rights framework to address the issues raised by the applications of neurotechnologies. In other words, the objective of this program is to assess whether the fundamental ethical-legal issues raised by neurotechnology “can be sufficiently addressed by the existing human rights framework or whether new human rights pertaining to cognitive liberty, mental privacy, and mental integrity and psychological continuity, need to be entertained in order to govern neurotechnologies.” In parallel, national legislators are also active in the area of neurotechnology governance. At the level of national legislation, the most important policy development in this area is the recent approval by the Chilean Senate of a constitutional reform law that defines mental integrity as a fundamental human right, and a law on neuroprotection that protects brain data and applies existing medical ethics, codified in the current Chilean medical code, to the use of neurotechnologies in non-patient populations. This makes Chile, as noted by Yuste et al. (2021) “the only country with a proposed law and constitutional amendment mandating neuroprotection and explicitly protecting neurorights.” Furthermore, the Spanish Secretary of State for AI has recently published a Charter of Digital Rights that incorporates neurororights as part of citizens’ rights for the new digital era. Finally, the Italian Data Protection Authority has devoted the 2021 Privacy Day to the investigation of neurorights and endorsed their necessity to properly addressing the implications of neurotechnology for human rights, especially privacy rights.

## Open Questions and the Future of Neurorights

Although (or perhaps precisely because) neurorights have moved in a relatively short time from the domain of ethical-legal reflection to that of advocacy and policy, many questions still remain unanswered. The first question is to determine whether neurorights should interpreted as rights in the philosophical sense (moral rights), as rights in the sense of international human rights law (legal rights) or all the above.

The second and most pressing question is to determine whether neurorights in the sense of international human rights law are to be interpreted as brand new human rights or as evolutionary interpretations of existing rights. Two problem-solving principles may offer guidance in this regard. First, as we have seen, Occam’s razor or law of parsimony requires that entities should not be multiplied without necessity. Second, the principle of avoiding “rights inflation,” i.e., the objectionable tendency to label everything that is morally desirable as a “human right,” postulates that the unjustified proliferation of new rights should be avoided. The unjustified proliferation of human rights is problematic because it may spread skepticism about all human rights, as it dilutes them to mere moral desiderata or purely rhetorical claims. In other words, rights inflation is to be avoided because it dilutes the core idea of human rights and distracts from the central goal of human rights instruments, which is to protect a set of truly fundamental human interests, and not everything that would be desirable or advantageous in an ideal world.

From this perspective, the most parsimonious approach would be considering neurorights by default as evolutionary interpretations of existing rights, while at the same time imposing justificatory tests to assess whether they actually constitute new human rights. Several justificatory tests to prevent rights inflation have been proposed. For example, Alston (1984) proposed a list of criteria that a normative claim must satisfy in order to qualify as a “human right.” In his view, the new human right candidate must (i) “reflect a fundamentally important social value”; (ii) “be consistent, but not merely repetitive, of the existing body of international human rights law”; (iii) “be capable of achieving a very high degree of international consensus,” and (iv) “be sufficiently precise as to give rise to identifiable rights and obligations” (Alston, 1984). Similarly, Nickel has required that a proposed human right should not only (i) deal with some very important good but also (ii) respond to a common and serious threat to that good, (iii) impose burdens on the addressees that are justifiable and no larger than necessary, and (iv) be feasible in most of the world’s countries (Nickel et al., 2013).

A third question regards how neurorights can be adequately implemented and enforced. If in the future some of the neurorights described in this paper were to pass several justificatory tests and obtain strong democratic and deliberative support, how should they be enforced? There are two types of human rights instruments: declarations and conventions. Declarations are not legally binding but do have political impact, whereas conventions are legally binding under international law. Both declarations and conventions can become customary international law over time, which makes them universally legally binding (Moscrop, 2014). Future legal scholarship should discuss which type of instrument is most suitable for enshrining neurorights into international human rights law. Further, it should determine how the problem of “under-enforcement” of human rights can be avoided (Koh, 1998), that is how to achieve state obedience of neurorights laws from a realist perspective.

As the analysis above attests, for the field of neurorights to progress and have consistent impact on policy, it would need to overcome the current semantic variations and ambiguities in how these neurorights are denominated, defined, and interpreted. Without a common terminology, semantic disambiguation and conceptual harmonization, it is unlikely that neurorights-based initiatives will lead to effective national and international policies. This harmonization process should not obliterate divergent views, but include them in a pluralistic and deliberative democratic manner. However, it should ensure that neuroright proposals are adequately vetted, conceptually demarcated, normatively justified and rooted in both moral philosophy and existing regulations.

Finally, future scholarship should discuss the place of neurorights within the governance of neurotechnology. Unless one commits to the unlikely thesis that neurorights are sufficient for neurotechnology governance (hence that neurotechnology governance can be entirely reduced to neurorights promotion), it is critical to clarify how neurorights relate to other governance mechanisms, such as self-regulation by neurotechnology actors, ethical guidelines, and binding regulations in areas, such as *inter alia* health law, data protection law, consumer protection law and criminal law.

## Conclusion

The above analysis suggests that neurorights reflect fundamental human interests that are deeply rooted in the history of ideas. These rights introduce normative specifications related to the protection of the person’s mental and neural domain that are not merely repetitive of existing human rights frameworks. Moreover, it corroborates the view that the fundamental rights and freedoms relating to the human mind and brain are the fundamental substrate of other rights and freedoms. Therefore, protecting neurorights is a fundamental task of international human rights law and may contribute to expanding the protection of other rights and freedoms.

This overview indicates that there is still no complete consensus regarding the conceptual-normative boundaries and terminology of neurorights. Divergences exist in relation to how these rights are interpreted, formulated, and conceptually articulated. However, a certain degree of convergence is emerging around three families of neurorights.

First, the right to mental integrity appears to have the highest degree of theoretical consensus and legal entrenchment. This is because it is already enshrined in international human rights law and provides a solid legal framework that prioritizes protection from harm. Second, the need for specific provisions on the protection of private mind-related information (through mental privacy and neuroprivacy) also seems to share a high degree of acceptance and recognition. Three, a variety of neurorights candidates have been proposed to preserve and promote the freedom of the human mind and thereby prevent external manipulation. These include evolutionary interpretations of the right to freedom of thought, the right to cognitive liberty, and the right to personal identity. This third family of neurorights is of foundational importance as it largely considered a substrate of all other neurorights and derived freedoms.

The three families of neurorights above appear deeply rooted in the history of philosophy, international human rights frameworks, and legal doctrine. However, they are affected by several challenges. First, neurorights are insufficiently specified in current human rights instruments, such as the UDHR, the ECHR and the CFR. For example, although it is enshrined within Article18(1) of the ICCPR, the scope and content of “freedom of thought” is largely underexplored. The same can be said for the notion of “mental integrity” as addressed in the CFR. Therefore, a process of either normative interpretation or reform appears to be needed to adequately specify the principles of freedom or entitlement related to a person’s mind and brain domain in the digital era. Further research is needed to investigate the novel challenges for freedom of thought posed by emerging technologies, such as neurotechnologies and AI and clarify the relationship between the protection of the *forum externum* (that is, protecting the manifestations or externalizations of thought, such as religion, belief, and expression) and the protection of the *forum internum* (that is, protecting thought itself). Further research is also needed to explore the relationship between freedom of thought and the bundle of rights that fall under the cognitive liberty domain. Clarifying the status of brain data and mental information from a data protection perspective is necessary to define the scope of the right to mental privacy. Finally, the notion of manipulation—which is often invoked as a risk scenario to which the rights to freedom of thought, mental integrity and cognitive liberty could respond—appears elusive to a clear definition, hence requires further analysis to clearly determine the conditions for (il)legitimate influence into a person’s mind. The results of the Council of Europe’s Strategic Action Plan on human rights and technologies in biomedicine as well as an upcoming report to the UN General Assembly on “Freedom of Thought” may help address these challenges.

Normative evolution in the light of disruptive technological innovation is not unprecedented in the history of science. For example, the development of the mechanical ventilator produced the concept of brain death and required the law to specify more clearly what functions are integral to life and what are not (Machado, 2007). Similarly, advances in genetic sequencing and genome editing have led to novel human right instruments related to genetics, such as the 1997 Universal Declaration on the Human Genome and Human Rights (UDHGHR) and the 2003 by the International Declaration on Human Genetic Data (IDHGD). These instruments also introduced new rights, such as the “right not to know one’s genetic information” [UDHGHR Art. 5(c); IDHGD (Art. 10)].

It is desirable that neurorights shall follow a similar historical trajectory in a manner that expands and enhances the capacity of our human rights framework to address the profound implications of neurotechnology and AI for human nature, human dignity, and human rights.

## Author Contributions

The author confirms being the sole contributor of this work and has approved it for publication.

## Conflict of Interest

MI is a member of the Council of the Neurorights Network and a member of the Steering Committee on Neurotechnology of the OECD and has served as an expert advisor to the Council of Europe’s Ad Hoc Committee on Artificial Intelligence on the topic of AI & human rights. He is currently serving as an expert advisor to the Council of Europe’s Committee on Bioethics as part of the Strategic Action Plan on Human Rights and Technologies in Biomedicine.

## Publisher’s Note

All claims expressed in this article are solely those of the authors and do not necessarily represent those of their affiliated organizations, or those of the publisher, the editors and the reviewers. Any product that may be evaluated in this article, or claim that may be made by its manufacturer, is not guaranteed or endorsed by the publisher.

## Footnotes

1 Unlike usually assumed, the Latin phrase “primum non-nocere” is not of ancient origin. Smith (2005) traced it back to an attribution to Thomas Sydenham (1624–1689) in a book by Inman (1861) entitled *Foundation for a New Theory and Practice of Medicine.* See: Smith (2005). Origin and Uses of Primum Non-Nocere—Above All, Do No Harm! *The Journal of Clinical Pharmacology* 45, 371–377.

2 See: *Goodwin v the UK* (2002) 35 EHRR 18 at 90.

3 A keyword search of “neurorights” in the Google search engine retrieved over 22,000 results. The same keyword search in Google Scholar retrieved slightly more than 100 entries (stand: June 2021).
